# Effects of Multicomponent and Multiprofessional Interventions on Cardiovascular and Functional Health in Hypertensive and Normotensive Older Women: A Case Study

**DOI:** 10.3390/jcm15020572

**Published:** 2026-01-10

**Authors:** Jordan Hernandez-Martinez, Pablo Valdés-Badilla, Izham Cid-Calfucura, Edgar Vásquez-Carrasco, Marilene Ghiraldi de Souza Marques, Braulio Henrique Magnani Branco

**Affiliations:** 1 Department of Physical Activity Sciences, Universidad de Los Lagos, Osorno 5290000, Chile; jordan.hernandez@ulagos.cl; 2 Department of Education, Faculty of Humanities, Universidad de la Serena, la Serena 1700000, Chile; 3 Department of Physical Activity Sciences, Faculty of Education Sciences, Universidad Católica del Maule, Talca 3530000, Chile; 4 Sports Coach Career, Faculty of Life Sciences, Universidad Viña del Mar, Viña del Mar 2520000, Chile; 5 Department of Physical Activity, Sports and Health Sciences, Faculty of Medical Sciences, Universidad de Santiago de Chile (USACH), Santiago 8370003, Chile; izham.cid@gmail.com; 6 School of Occupational Therapy, Faculty of Psychology, Universidad de Talca, Talca 3465548, Chile; edgar.vasquez@utalca.cl; 7 Centro de Investigación en Ciencias Cognitivas, Faculty of Psychology, Universidad de Talca, Talca 3465548, Chile; 8 VITALIS Longevity Center, Universidad de Talca, Talca 3465548, Chile; 9 Graduate Program in Health Promotion, Cesumar University (UniCesumar), Maringá 87050-900, Brazil; marileneghiraldi@gmail.com (M.G.d.S.M.); braulio.branco@unicesumar.edu.br (B.H.M.B.)

**Keywords:** biomarkers, cardiovascular system, body composition, physical functional performance, healthy aging

## Abstract

**Background/Objectives:** This study aimed to examine the effects of changes over time during multicomponent training (MCT) combined with multiprofessional interventions at different time points [baseline (T0), 12 weeks (T1), 24 weeks (T2) and 36 weeks (T3)] on body composition; blood pressure (SBP and DBP); biomarkers [fasting glucose, total cholesterol, high-density lipoprotein (HDL-c), low-density lipoprotein (LDL-c), and triglycerides]; and physical improvement [maximal isometric handgrip strength (MIHS), arm curl, 30 s chair stand, six-minute walk test (6MWT), and timed up-and-go (TUG)] in hypertensive and normotensive older women. **Methods:** This longitudinal and experimental study was conducted in hypertensive (*n* = 23, mean age 69.7 ± 7.21 years) and normotensive (*n* = 17, mean age 71.3 ± 5.92 years) older women, with three 90 min sessions per week for 36 weeks, including 60 min of MCT, 30 min of nutritional education (twice a week) and 30 min of psychoeducation (once a week). **Results:** Significant decreases in SBP at T1 and T3 and DBP at T3 were detected in both groups, and only SBP at T2 was detected in normotensive women (*p* < 0.05). Significant reductions in fasting glucose at T1-T2-T3 and LDL-c and total cholesterol at T3 and triglycerides at T2 were detected in hypertensive patients (*p* < 0.05). Significant improvements in arm curl at T1 and the 30 s chair stand at T1–T3 were observed for both groups, and improvements at T2–T3 were detected only in hypertensive patients (*p* < 0.05). **Conclusions:** MCT and multiprofessional interventions improve blood pressure, biomarkers and physical improvement in hypertensive and normotensive older women.

## 1. Introduction

Cardiovascular diseases are among the leading causes of death in 32% of the world’s population [1], with high blood pressure being one of the leading causes of premature death among people aged 30–79 years worldwide [2]. In South America, the prevalence of high blood pressure is between 20% and 30%, with the highest rates in Brazil [3]. It affects mainly women, accounting for 52.3% of cases in urban areas of the country (86.2%), mainly in the southeast region (43.9%) [4]. There is a significant association (*p* < 0.01) between high blood pressure and high serum levels of total cholesterol, low-density lipoprotein (LDL-c), triglycerides, and fasting glucose, as well as low levels of high-density lipoprotein (HDL-c), increasing the risk of overweight and obesity [5], along with a decrease in physical fitness, which affects the quality of life of older people [5,6].

Therefore, implementing interventions that help counteract these changes in health indicators in older people with hypertension is important [7]. An efficient non-pharmacological treatment for enhancing the health of older people with hypertension is physical activity and exercise [8]. However, the response to physical exercise interventions may vary depending on the type of training [9] and its duration [10]. In a meta-analysis conducted by Hejazi et al. [11] in hypertensive older people, significant decreases (*p* < 0.01) in systolic (SBP) and diastolic (DBP) blood pressure were observed through aerobic training, resistance training, and concurrent training, whereas in the lipid profile, there were significant decreases (*p* < 0.05) in total cholesterol and LDL-c through concurrent training and resistance training, along with a significant increase (*p* < 0.01) in HDL-c only in concurrent training and aerobic training. In a quasi-experimental study conducted by Coelho Junior et al. [12] in hypertensive and normotensive older people using an MCT intervention of 6 months, significant improvements (*p* < 0.05) in physical improvement were observed in the walking speed and one-leg stand tests in hypertensive older people, with no significant improvements in the Timed Up-and-Go (TUG) test in either group. In a randomized controlled trial conducted by Leitão et al. [13] in normotensive older women, there were significant decreases (*p* < 0.05) in total cholesterol and triglyceride levels in favour of those in the MCT group compared with those in the inactive control group. Forte et al. [14] reported that, compared with that in the inactive control group, the body fat percentage (BFP) in normotensive older people in favour of MCT was significantly lower (*p* = 0.04).

Although the benefits of MCT are well documented [12,15], its combined effects with multiprofessional interventions on biomarkers and functional improvement in hypertensive versus normotensive older women remain unclear. Similarly, the response to different intervention times (duration in weeks) is unknown, which may affect the response to the intervention [16]. Likewise, the response to exercise interventions may vary depending on blood pressure levels (hypertensive and normotensive) [17]. Therefore, this study aimed to examine time-related changes in health-related outcomes during a multicomponent training (MCT) programme combined with multiprofessional interventions (nutritional education and psychoeducation) in hypertensive and normotensive older women. Health outcomes included body composition [BFP and fat-free mass (FFM)], blood pressure (SBP and DBP), cardiometabolic biomarkers (fasting glucose and lipid profile), and physical improvement measures (maximal isometric handgrip strength, arm curl, 30 s chair stand, 6 min walk test, and TUG). It was hypothesized that participation in the MCT programme combined with multiprofessional interventions would be associated with favourable changes over time in cardiovascular variables (SBP and DBP), selected cardiometabolic biomarkers (fasting glucose and lipid profiles), and physical fitness, particularly muscle strength, with potentially different magnitudes of response according to baseline blood pressure status [12,15].

## 2. Materials and Methods

### 2.1. Study Design

This study employed an analytical, experimental, longitudinal, repeated-measures design with a quantitative approach. Participants were non-randomly allocated to two parallel groups on the basis of blood pressure status: hypertensive (SBP ≥ 130 mmHg or DBP ≥ 80 mmHg) and normotensive (SBP < 130 mmHg and DBP < 80 mmHg). CONSORT standards and a previous study [18] were used as the methodology. Additionally, the study was prospectively registered in relation to participant enrolment, and the study outcomes and statistical analysis plan were conducted in accordance with prespecified protocols. The trial has been registered at the United States’ ClinicalTrials.gov (code: NCT07152158; https://register.clinicaltrials.gov/prs/beta/studies/S000G6TG00000042/recordSummary, accessed on 1 December 2025), obtained on 22 August 2025. The interventions lasted 36 weeks (108 sessions) at four time points (T0: baseline; T1: 12 weeks; T2: 24 weeks; and T3: 36 weeks). There were three weekly sessions (Mondays, Wednesdays, and Fridays) that lasted 90 min each. The interventions included MCT (60 min three times a week), nutritional education (30 min twice a week), and psychoeducation (30 min once a week). The present study was publicized through various communication channels (e.g., television, radio, social media) to recruit participants. Body composition (BFP and FFM), blood pressure (SBP and DBP), biochemical variables (fasting glucose, total cholesterol, HDL-c, LDL-c, and triglycerides), and physical fitness (MIHS, arm curl, 30 s chair stand, 6MWT, and TUG) were assessed. All measurements were taken in the morning, between 9:00 and 11:00 a.m., and in the same place: the Interdisciplinary Laboratory for Health Promotion Interventions (LIIPS) at Cesumar University, Maringa, Paraná, Brazil. Moreover, the training sessions (MCT) along with nutritional education and psychoeducation were held between 8:00 a.m. and 9:30 a.m. The older women reported no pain prior to the evaluations or throughout the training sessions, and they did not have any musculoskeletal or cardiorespiratory problems during the intervention. Participant adherence to the intervention was rigorously monitored through attendance lists for all scheduled sessions. The overall adherence rate for the hypertensive older women was 87% ± 2%, and for the normotensive older women, it was 89% ± 3%. Given that the daily sessions always commenced with the theoretical (nutritional or psychoeducational) component immediately followed by the practical (MCT) component, adherence was consistent across all daily activities. Although the study did not exhaustively monitor every intercurrent event (e.g., minor illnesses, holidays), participants were instructed to report any absences or significant health issues. To optimize the ecological validity of the study within a practical application context, participants were instructed to maintain their habitual medication routines and dietary patterns without study-induced modifications. Any self-reported changes in medication, including type, dosage, and start/end dates of antihypertensive or lipid-lowering drugs, or significant health events were recorded by the study team, although a formal, systematic control or quantification of medication adherence and dietary variability across all participants was beyond the scope of this quasi-experimental design. This approach aims to simulate a community intervention setting, acknowledging the inherent limitations of rigorous control over such variables. Beyond the scheduled sessions, there was no direct monitoring of other external physical activities.

### 2.2. Participants

The intervention started with 63 older women. According to the sample size calculation, 40 participants was the optimal number to confirm significant differences between the groups and time measurements. Statistical power was calculated using GPower software (version 3.1.9.6, Franz Faul, University of Kiel, Kiel, Germany). With an alpha threshold of 0.05, an 85% power, and a 15% anticipated loss, an initial investigation [19] showed that the least difference needed for significant clinical significance in a 30 s chair stand test was a mean difference of 0.46 replicates, with a standard deviation of 3.38 replicates.

**Figure 1:**
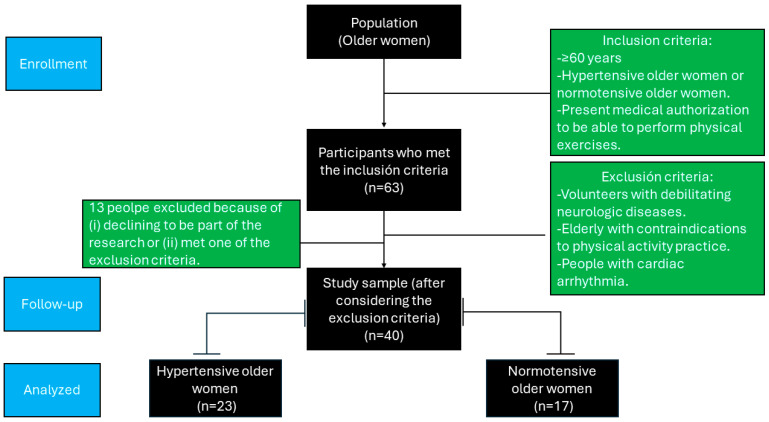
Flowchart of the recruitment process.

According to the following inclusion criteria, 63 people were invited to participate in the study: they had to be 60 years of age or older, have medical clearance to engage in physical activity, be able to understand and follow basic instructions in a contextualized manner, and score at least 24 on the Mini-Mental State Examination [20]; and were able to meet the intervention’s attendance requirement of at least 85%. The exclusion criteria were individuals who were diagnosed with debilitating neurological disorders (e.g., Alzheimer’s disease or Parkinson’s disease), older people with contraindications to physical exercise, and those with cardiac arrhythmias. Twenty-one older women either declined participation or did not meet the eligibility criteria. As a result, 40 participants were included in the study, comprising 23 hypertensive (mean age of 69.7 ± 7.21 years) and 17 normotensive older women (mean age of 71.3 ± 5.92 years) (Figure 1).

By signing an informed consent form, each participant gave permission for the use and processing of their personal data for scientific reasons. Importantly, all participants were legally competent in providing their own informed consent, and no consent from guardians or legal representatives was needed. The study was approved by the local Research Ethics Committee of Cesumar University (approval number: 3.373.307) in accordance with the guidelines set forth in Resolution 466/12 of the Brazilian Ministry of Health and the Declaration of Helsinki.

### 2.3. Blood Pressure

The older woman sat in a comfortable chair for 15 min in a quiet room. After this period, a cuff was placed on the midpoint of the upper left arm (heart level). An automatic blood pressure monitor (model HEM-7121, Omron, Osaka, Japan) was used to measure SBP and DBP. During blood pressure recording, the participants remained relaxed in the seated position, with their feet parallel to the width of their shoulder, with both their forearms and hands on the table, with their hands supinated, and with their backs against the chair, without moving or talking. The older women did not have access to blood pressure values during the measurement. The assessments lasted approximately 80 s and were performed three times with a one-minute rest between assessments. The mean of each person’s measurements was used in the final analysis. The cut-off points were as follows [21]: hypertension, SBP ≥ 130 mmHg and DBP ≥ 80 mmHg; normotensive, SBP < 130 mmHg and DBP < 80 mmHg. The measurements were collected on two separate days under identical conditions from the study participants, demonstrating a test–retest reliability of 0.99. Table 1 shows the baseline characteristics of the groups analysed.

### 2.4. Body Composition

The standing height of the participants was measured via a stadiometer (Welmy R-110^®^, Santa Bárbara D’Oeste, São Paulo, Brazil) attached to a scale with a maximum capacity of 2.2 m and an accuracy of 0.1 cm. Body composition was assessed via a tetrapolar bioelectrical impedance analyser (InBody 570^®^, Bio space Co. Ltd., Seoul, Republic of Korea), with a capacity of 250 kg and an accuracy of 100 g, following the manufacturer’s instructions and establishing recommendations to increase precision and reliability [22]. The following variables were assessed: body weight (kg), BFP, FFM (kg), and body mass index (BMI, kg/m^2^).

### 2.5. Biomarkers

The blood collection procedures followed the guidelines of the Clinical Laboratory Standards Institute [23]. The participants were previously instructed to prepare accordingly for the collections, which were conducted at the Clinical Analysis Laboratory of the Institution’s facilities. After the blood was drawn, pressure was applied to the puncture site to prevent bruising. The blood samples were distributed into the following tubes: Vacuplast^®^ collection tubes, tubes containing the anticoagulant ethylenediaminetetraacetic acid (EDTA) K2, and tubes with fluoride/EDTA anticoagulant. To obtain serum and plasma, the samples containing the fluoride/EDTA activator were centrifuged in a CENTRILAB^®^ analogue centrifuge at 3500 rpm (relative centrifugal force) for 15 min at room temperature. The following laboratory parameters were analysed: fasting glucose and lipid profile (triglycerides, HDL-c), LDL-c, and total cholesterol. Analyses were performed via Gold Analisa diagnostic kits (Belo Horizonte, Minas Gerais, Brazil) on the URIT 8021^®^ semiautomated biochemical and turbidimetric analyser by MHLab. All analyses were conducted in triplicate by a biomedical team blinded to the type of group intervention.

### 2.6. Physical Fitness Tests

#### 2.6.1. Maximal Isometric Handgrip Strength (MIHS)

MIHS testing has been suggested by earlier research [24]. It was found that sitting with the forearm and wrist in a neutral position, the elbow flexed at a 90-degree angle and kept near to the body, the spine correctly aligned, and the shoulder relaxed in a neutral position was the best testing position. The measurements were made using a handheld dynamometer (Jamar^®^, PLUS+, Sammons Preston, Patterson Medical, Warrenville, IL, USA). The dynamometer was adjusted to the first position, permitting contact between the thumb and the proximal phalanx of the index finger, in order to accommodate different hand sizes and guarantee effective engagement of the metacarpophalangeal and interphalangeal joints. In between each of the three hand repetitions, the participants were allowed to rest for 120 s.

#### 2.6.2. Arm Curl Test

In the arm curl test, participants sat on a chair and were asked to flex the elbow of their stronger arm as many times as possible in 30 s while holding a 2 kg dumbbell. The participants were instructed to maintain a normal breathing pattern and keep their elbow on their side during the entire test. A single repetition consisted of full elbow flexion from maximal extension [25].

#### 2.6.3. 30 s Chair Stand

To find the best improvement on the 30 s chair stand test, three repetitions were assessed [25]. It was performed while sitting in a chair with the arms resting across the chest for 30 s in order to assess the muscle strength of the lower limbs.

#### 2.6.4. 6 min Walking Test (6MWT)

Heart rate (HR) was recorded at rest and at the end of each minute during the 6MWT via a lightweight telemetric HR monitor (Polar FT1, Kempele, Finland). Oxygen saturation (SpO_2_) was recorded before and after completing the 6MWT via an oximeter and a finger sensor (Artery Check SB210, MD, Curitiba, Brazil). At the end of the test, distance was measured, and where appropriate, leg fatigue was assessed via a Borg scale ranging from 6–20 [26].

#### 2.6.5. Timed Up-and-Go (TUG) Test

Following earlier suggestions, the TUG test was conducted [25]. The person must leave an arm-supported chair, cross a three-meter aisle, turn around, and return to the chair. They must perform three trials and record the best one in seconds. A pair of evaluators measured the time using single-beam photocells (Brower Timing System, Draper, UT, USA), and the best of three trials was utilized for statistical analysis.

### 2.7. Interventions

The intervention activities consisted of three weekly MCT sessions and one session of nutritional and psychological education. The MCT intervention sessions lasted approximately 60 min and were conducted at university facilities. The exercises were designed to improve flexibility, strength, mobility, motor coordination, balance, and cardiorespiratory fitness. Notably, the MCT program was implemented alternately in indoor and outdoor settings three times a week, i.e., one day of the week was devoted to indoor training (gym setting), whereas another day was devoted to outdoor training (open space), alternating focusing on exercises with the respective body mass of the participants.

The MCT protocol consisted of an initial three-week period of neuromotor adaptation characterized by low volume and intensity. In the subsequent weeks, a gradual progression of volume and intensity was implemented, as illustrated in Table 2, following a classic linear periodization model [27,28]. Within the three weekly MCT sessions, the primary focus for strength exercises targeting large muscle groups, cardiorespiratory training (on treadmill, stationary bicycle, or rowing ergometer, according to participants’ preferences and physical condition), and balance or flexibility exercises was distributed across all sessions, with two sessions per week specifically emphasizing strength and cardiac components and the third often focusing on neuromotor coordination and flexibility elements. This flexible integration aimed to provide a comprehensive stimulus while allowing for individual adaptation. The training loads were adjusted throughout the intervention on the basis of perceptual and self-selected effort. The Rating of Perceived Exertion (RPE) scale, as described by Foster et al. [29], was employed to quantify and guide the intensity of each training session. This approach, which is widely used in older populations, promotes self-regulation of effort, enhances safety, and allows for individualized load adjustments on the basis of daily physical and perceived conditions, which is crucial for adherence and injury prevention. Before each training session, the Rating of Perceived Recovery (RPR) scale, proposed by Laurent et al. [30], was used to assess the individual’s recovery status relative to the previous session. All volunteers received prior instructions on how to use the perceptual scales during a briefing prior to the start of the training program. SpO_2_ and blood pressure (SBP and DBP) were measured before (initial) and after (final) each training session. Additionally, the volunteers were instructed to report any signs of chest discomfort, extreme fatigue, excessive sweating, or shortness of breath spontaneously. SpO_2_ was continuously monitored to detect potential hypoxia; if any participant presented with SpO_2_ < 88%, the exercise session was immediately discontinued.

The nutritional education component was based on the research by da Silva Oliveira and Silva-Amparo [31] with the aim of educating older women on healthy eating and dietary habits. These sessions were delivered exclusively in a group format, focusing on food literacy regarding labels and food processing; physiological processes; quality of life; and the importance of reducing risks associated with NCDs [32,33]. Importantly, no individualized meal plans or strict calorie or protein monitoring were provided to the participants. This approach was deliberately chosen to empower older women with knowledge and skills for self-management of health, promoting sustainable changes rather than adherence to restrictive regimens. The focus was on broad health promotion, consistent with public health goals, and aimed at enhancing the general understanding of macronutrients, micronutrients, hydration, sodium management, and meal planning strategies for older people. Nutritional education sessions were conducted two times a week, in a group format, prior to the MCT sessions, for 30 min. Adherence to these sessions was monitored via attendance lists. The structured curriculum included the following themes: orientation and individualized goal-setting weight management principles for older people; protein quality, leucine, and distribution across meals; carbohydrate quality, fibre, and glycemic control; dietary fats, omega-3 s, and cardiovascular health; sodium reduction and blood pressure management; hydration and fluid balance (including diuretic considerations); calcium, vitamin D, and bone health; vitamin B12; folate, iron, and anemia prevention; potassium, magnesium, and cardiometabolic support; chronic kidney disease nutrition basics; heart failure, fluid and sodium strategies; meal planning, plate method and portion sizes; label reading, including added sugars, sodium, and trans fats; smart breakfasts and protein-rich snacks; cooking skills, including low-sodium; high-flavour techniques; budget-friendly shopping; pantry, and frozen staples; food safety and safe storage for seniors; oral health; dysphagia; texture modifications; gastrointestinal health, including constipation, reflux, and fibre–fluid balance; mindful eating and appetite regulation; emotional eating; stress, and coping skills; sleep quality and metabolic health; physical activity synergy, strength, and protein timing. While no formal assessment of changes in dietary knowledge or habits was conducted as a primary or secondary outcome, participant engagement and qualitative feedback were noted.

The psychoeducation component involved structured weekly group sessions, conducted once a week, prior to the MCT sessions, for 30 min. It was based on therapeutic interventions aimed at providing knowledge and fostering the potential for change in light of the psychological consequences of the aging process within a treatment and prevention model for psychiatric disorders [34]. The curriculum, structured as a progressive syllabus, covered themes such as group introduction, objectives, ground rules, and therapeutic contracts, mental health and the biopsychosocial model, stress: physiology, triggers, and window of tolerance, thought emotion behavior links, identifying and labelling emotions, cognitive distortions and basic restructuring, mindfulness: applying present moment awareness to daily life, emotion regulation: distress tolerance; behavioral activation and routine planning, personal values and smart goals, habit formation, motivation, and adherence, step solving, and stress-related strategies; stress: safety, stabilization, and grounding; grief and loss: tasks and social support; chronic pain and mind body integration; substance use and harm reduction; eating, mood, emotional hunger, anger, impulsivity, and sleep hygiene; anxiety: psychoeducation and gradual exposure; depression: inactivity cycle and coping; mood disorders: warning signs and monitoring; psychosis: early recognition and grounding strategies; trauma: safety, stabilization, and grounding; grief; and loss: tasks and social support; chronic pain and mind body integration; substance use and harm reduction; eating, mood, emotional hunger; anger, impulsivity; and de-escalation. While formal psychometric outcomes were not assessed as primary or secondary endpoints of this study, participant engagement and qualitative feedback during discussions were observed by the facilitating psychologists, suggesting perceived value from the intervention.

### 2.8. Statistical Analysis

GraphPad Prism version 9.0 statistical software was used to analyse descriptive and inferential data. The descriptive statistics included the calculation of the mean, standard deviation and confidence interval (95% CI). The delta (Δ) values were also calculated by subtracting the post-measurements from the pre-measurements for the variables that exhibited significant differences. The Shapiro–Wilk test was used to determine the data distribution. A two-factor mixed analysis of variance (ANOVA) model with repeated measures was subsequently performed to measure the time × group effects of BFP, FFM, fasting glucose, HDL-c, LDL-c, total cholesterol, triglycerides, MIHS-dominant and non-dominant hands, TUG, 6WMT, arm curl, and a 30 s chair stand. When the time × group interaction was significant, a Bonferroni multiple comparisons test (post hoc) was performed to establish intergroup (pre- vs. post-assessments) and intragroup (pre vs. 12 weeks vs. 24 weeks vs. 36 weeks; and older women hypertensive vs. older women normotensive) differences. The partial eta squared (ηp^2^) was computed to ascertain the effect size of the time × group interaction. The results were interpreted taking into account ηp^2^ values of 0.01, 0.06, and 0.14, which correspond to modest, moderate, and high effect sizes, respectively [35]. Cohen’s d was used to compute the effect size for multiple comparisons, taking into account modest (≥0.2), moderate (≥0.5), or large (≥0.8) effects. An α value of 0.05 was taken into consideration for every analysis.

## 3. Results

### 3.1. Body Composition

**Figure 2:**
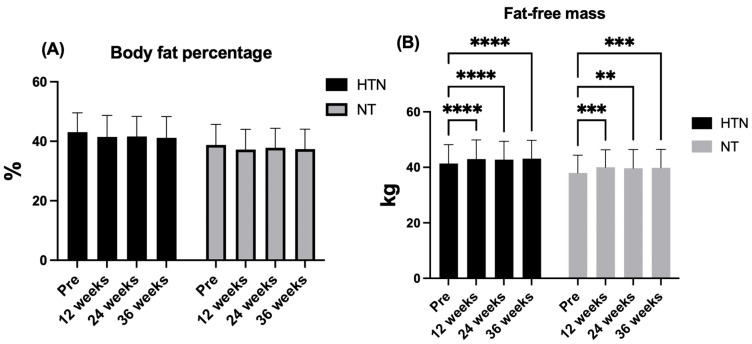
Changes in body fat percentage (**A**) and fat-free mass (**B**) across the intervention period in hypertensive (HTN) and normotensive (NT) older women. The data are presented as the means and (±) standard deviations. ** *p* < 0.01, *** *p* < 0.001, **** *p* < 0.0001. %: percentage; kg: kilograms.

Across the intervention period, body composition outcomes showed a divergent pattern between fat mass and lean mass adaptations. BFP did not change significantly in either hypertensive or normotensive older women. Accordingly, no significant time × group interaction was observed (F_1.30,49.47_ = 0.16; *p* = 0.76; η^2^p = 0.00; *small effect*), nor was there a main effect of group (F_1,38_ = 3.68; *p* = 0.063; η^2^p = 0.09; *moderate effect*), despite the presence of a significant main effect of time (F_1.30,49.47_ = 4.68; *p* = 0.026; η^2^p = 0.11; *moderate effect*). However, post hoc analyses did not reveal significant differences between baseline and any follow-up time point within either group or between groups at any time point (all adjusted *p* > 0.05). In contrast, FFM increased significantly over time in both groups. No time × group interaction (F_2.27,86.14_ = 0.50; *p* = 0.633; η^2^p = 0.01; *small effect*) or main effect of group (F_1,38_ = 2.28; *p* = 0.140; η^2^p = 0.06; *moderate effect*) was detected, whereas a robust main effect of time was evident (F_2.27,86.14_ = 43.70; *p* < 0.0001; η^2^p = 0.53; *large effect*). Post hoc analyses demonstrated significant increases in FFM at 12, 24, and 36 weeks compared with baseline in hypertensive older women (Δ = 1.40–1.74 kg; 95% CI: 0.71 to 2.38; all adjusted *p* < 0.0001), as well as in normotensive older women (Δ = 1.67–2.05 kg; 95% CI: 0.48–3.06; adjusted *p* = 0.0001–0.0049), with no significant differences between follow-up time points or between groups. Figure 2 shows the changes in the body composition variables in hypertensive and normotensive older women.

### 3.2. Biomarkers

With respect to biomarkers, HDL-c remained stable over time in both groups. No significant time × group interaction was observed (F_1.98,75.11_ = 0.34, *p* = 0.710, η^2^p = 0.01; *small effect*), nor was there a main effect of time (F_1.98,75.11_ = 2.90, *p* = 0.062, η^2^p = 0.07; *moderate effect*). In contrast, a significant main effect of group was detected (F_1,38_ = 11.94, *p* = 0.001, η^2^p = 0.24; *large effect*), with normotensive older women consistently exhibiting higher HDL-c levels than hypertensive older women at baseline (Δ = 11.24 mg/dL, 95% CI: 4.29–18.19; *p* = 0.002), 12 weeks (Δ = 10.07 mg/dL, 95% CI: 3.01–17.12; *p* = 0.007), 24 weeks (Δ = 8.48 mg/dL, 95% CI: 1.71–15.26; *p* = 0.016), and 36 weeks (Δ = 11.96 mg/dL, 95% CI: 2.27–21.64; *p* = 0.017). Post hoc analyses confirmed these differences at all time points (all *p* ≤ 0.017), with large between-group effect sizes (baseline *d* = 1.07; 12 weeks *d* = 0.93; 24 weeks *d* = 0.78; *moderate effect*; 36 weeks *d* = 0.87; *large effect*).

LDL-c exhibited a significant effect of time (F_2.66,101.0_ = 5.43, *p* = 0.0078, η^2^p = 0.10; *moderate effect*), whereas no time × group interaction (F_2.66,101.0_ = 0.31, *p* = 0.790, η^2^p = 0.01; *small effect*) or main effect of group (F_1,38_ = 0.87, *p* = 0.357, η^2^p = 0.02; *small effect*) was detected. Post hoc analyses indicated significant reductions at 24 weeks (Δ = −12.50 mg/dL, 95% CI: −24.52 to −0.49; *p* = 0.039) and 36 weeks (Δ = −17.47 mg/dL, 95% CI: −31.08 to −3.86; *p* = 0.0087) compared with baseline in hypertensive older women only.

A comparable temporal pattern was observed for total cholesterol, with a significant main effect of time (F_2.72,103.2_ = 6.94, *p* = 0.0004, η^2^p = 0.15; *large effect*), in the absence of a time × group interaction (F_2.72,103.2_ = 0.13, *p* = 0.926, η^2^p = 0.00; *small effect*) or a main effect of group (F_1,38_ = 1.71, *p* = 0.199, η^2^p = 0.04; *small effect*). Post hoc analyses demonstrated significant reductions at 12 weeks (Δ = −16.04 mg/dL, 95% CI: −27.68 to −4.40; *p* = 0.0047), 24 weeks (Δ = −16.26 mg/dL, 95% CI: −28.90 to −3.63; *p* = 0.0085), and 36 weeks (Δ = −21.48 mg/dL, 95% CI: −33.06 to −9.89; *p* = 0.0002) relative to baseline in hypertensive older women. No significant within-group changes were observed in normotensive older women, and no significant between-group differences were detected at any time point.

For fasting glucose, no significant time × group interaction was found (F_2.78,105.6_ = 0.27, *p* = 0.794, η^2^p = 0.01; *small effect*), whereas significant main effects of time (F_2.78,105.6_ = 8.22, *p* = 0.0005, η^2^p = 0.15; *large effect*) and group (F_1,38_ = 4.55, *p* = 0.0386, η^2^p = 0.11; *moderate effect*) were observed. Post hoc analyses revealed significant reductions at 12 weeks (Δ = −5.17 mg/dL, 95% CI: −9.07 to −1.28; *p* = 0.0065), 24 weeks (Δ = −7.09 mg/dL, 95% CI: −12.38 to −1.80; *p* = 0.0061), and 36 weeks (Δ = −5.30 mg/dL, 95% CI: −10.42 to −0.19; *p* = 0.0401) compared with baseline in hypertensive older women, with no significant within-group changes in normotensive older women. Between-group differences were present at baseline and 12 weeks, with higher glucose levels in hypertensive older women and moderate to moderate–large effect sizes (baseline *d* = 0.61; *moderate effect*; 12 weeks *d* = 0.69; *moderate effect*).

**Figure 3:**
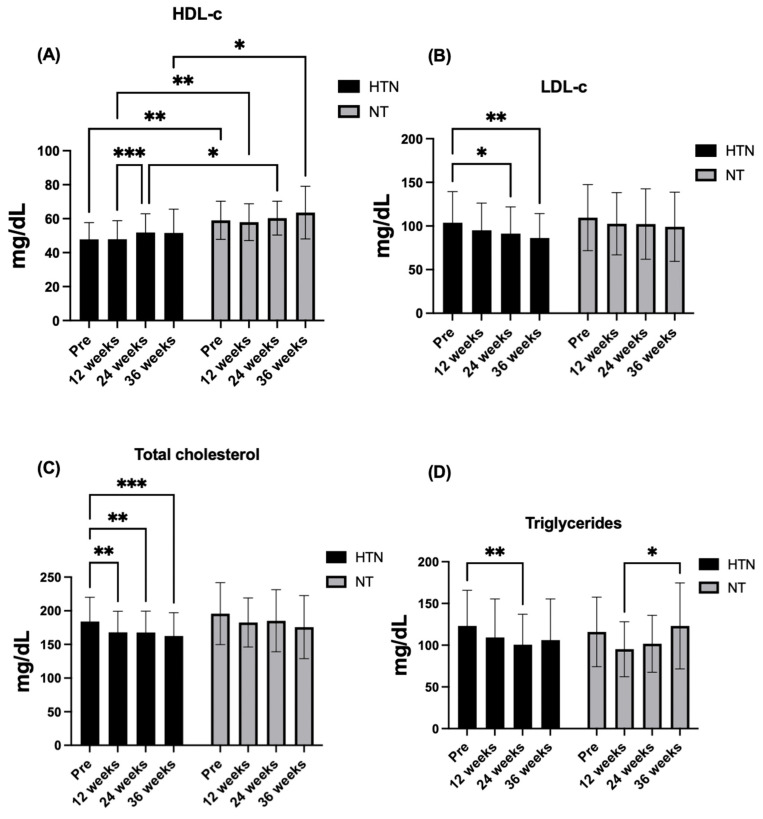
Changes in (**A**) HDL-c, (**B**) LDL-c, (**C**) total cholesterol, and (**D**) triglycerides across the intervention period in hypertensive (HTN) and normotensive (NT) older women. The data are presented as the means ± standard deviations. * *p* < 0.05, ** *p* < 0.01, *** *p* < 0.001. mg/dL: milligrams per deciliter.

**Figure 4:**
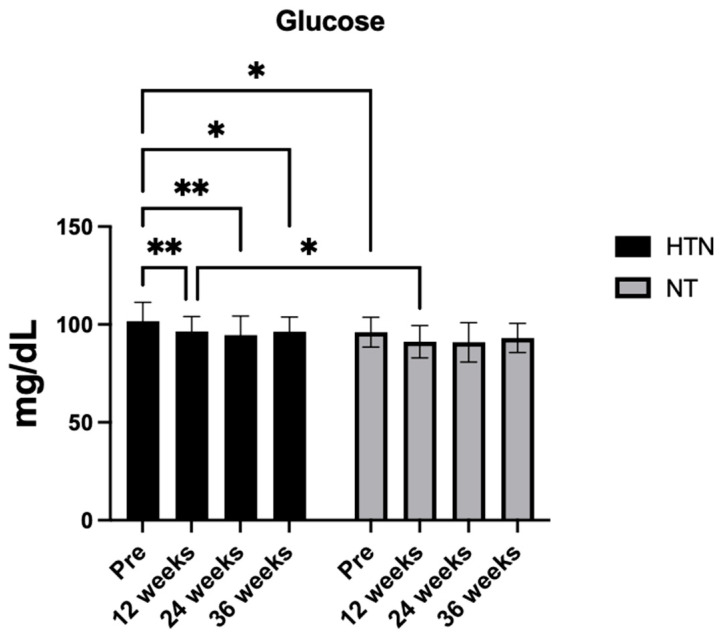
Changes in glucose across the intervention period in hypertensive (HTN) and normotensive (NT) older women. The data are presented as the means ± SDs. * *p* < 0.05, ** *p* < 0.01. mg/dL: milligrams per deciliter.

Finally, there was no significant time × group interaction (F_2.32,88.06_ = 2.60, *p* = 0.072, η^2^p = 0.06; *small effect*) or main effect of group (F_1,38_ = 0.004, *p* = 0.948, η^2^p = 0.00; *small effect*), but there was a significant main effect of time (F_2.32,88.06_ = 4.86, *p* = 0.0072, η^2^p = 0.11; *moderate effect*). Post hoc analyses indicated a significant increase at 24 weeks compared with baseline in hypertensive older women (Δ = +22.61 mg/dL, 95% CI: 7.89–37.33; *p* = 0.0017). Among normotensive older women, a significant difference was observed between 12 and 36 weeks (Δ = −27.82 mg/dL, 95% CI: −52.41 to −3.24; *p* = 0.0239), whereas no other within-group or between-group differences were detected. Figure 3 and Figure 4 show the changes in the biomarker variables in hypertensive and normotensive older women.

### 3.3. Blood Pressure

For SBP, significant main effects of time (F_2.61,99.18_ = 13.73, *p* < 0.0001, η^2^p = 0.27; *large effect*) and group (F_1,38_ = 31.64, *p* < 0.0001, η^2^p = 0.45; *large effect*) were observed in the absence of a time × group interaction (F_2.61,99.18_ = 0.81, *p* = 0.476, η^2^p = 0.02; *small effect*). Post hoc analyses indicated consistently lower SBP values in normotensive than in hypertensive older women at all time points (all adjusted *p ≤* 0.001), with large between-group effect sizes at baseline (*d* = 1.33; *large effect*), 12 weeks (*d* = 1.28; *large effect*), 24 weeks (*d* = 1.12; *large effect*), and 36 weeks (*d* = 1.16; *large effect*). Within-group analyses demonstrated significant SBP reductions at 12 weeks (Δ = −15.04 mmHg, 95% CI: −28.48 to −1.61; *p* = 0.024) and 36 weeks (Δ = −22.99 mmHg, 95% CI: −31.84 to −14.14; *p* < 0.0001) relative to baseline in hypertensive older women. Comparable reductions were observed in normotensive older women at 12 weeks (Δ = −17.94 mmHg, 95% CI: −32.45 to −3.43; *p* = 0.013) and 36 weeks (Δ = −16.35 mmHg, 95% CI: −29.91 to −2.80; *p* = 0.016).

**Figure 5:**
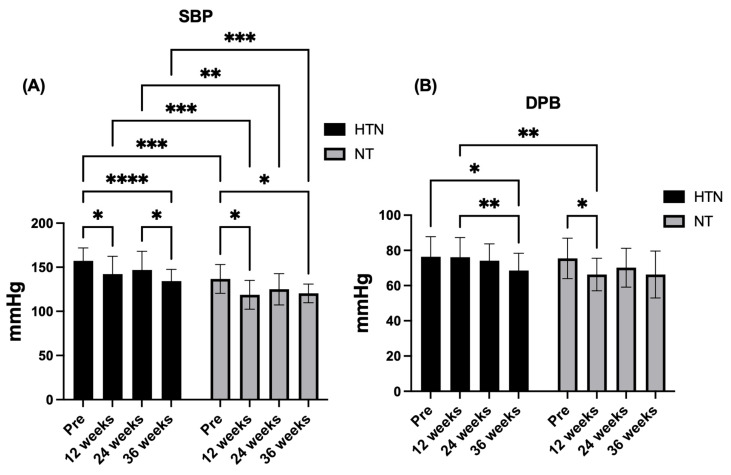
Changes in (**A**) SBP and (**B**) DBP across the intervention period in hypertensive (HTN) and normotensive (NT) older women. The data are presented as the means and (±) standard deviations. * *p* < 0.05, ** *p* < 0.01, *** *p* < 0.001. **** *p* < 0.0001. SBP: systolic blood pressure; DBP: diastolic blood pressure; mmHg: millimeters of mercury.

For DBP, no significant time × group interaction was detected (F_2.69,102.3_ = 2.13, *p* = 0.108, η^2^p = 0.05; *small effect*), nor was there a main effect of group (F_1,38_ = 2.64, *p* = 0.113, η^2^p = 0.06; *moderate effect*), whereas a significant main effect of time emerged (F_2.69,102.3_ = 6.71, *p* = 0.0006, η^2^p = 0.15; *large effect*). Post hoc analyses revealed a significant reduction in DBP at 36 weeks compared with baseline in hypertensive older women (Δ = −7.80 mmHg, 95% CI: −15.26 to −0.33; *p* = 0.038), as well as a significant difference between 36 and 12 weeks (Δ = −7.54 mmHg, 95% CI: −12.93 to −2.16; *p* = 0.004). In normotensive older women, DBP was significantly lower at 12 weeks than at baseline (Δ = −9.18 mmHg, 95% CI: −18.03 to −0.32; *p* = 0.041). A between-group difference was observed at 12 weeks only, favouring normotensive older women and accompanied by a large effect size (*d* = 0.97). Figure 5 shows the changes in the blood pressure variables in hypertensive and normotensive older women.

### 3.4. Physical Improvement

With respect to functional aerobic capacity, improvements in the six-minute walk test (6MWT) remained unchanged throughout the intervention. No significant time × group interaction (F_2.58,97.97_ = 2.53; *p* = 0.070, η^2^p = 0.06; *small effect*), main effect of time (F_2.58,97.97_ = 1.22; *p* = 0.305; η^2^p = 0.03; *small effect*), or group (F_1,38_ = 0.47; *p* = 0.498; η^2^p = 0.01; *small effect*) was observed. Post hoc analyses confirmed the absence of between-group differences at baseline (Δ = −1.15 m, 95% CI: −31.57–29.27; *p* = 0.939), 12 weeks (Δ = −7.65 m, 95% CI: −51.19–35.89; *p* = 0.722), 24 weeks (Δ = 14.74 m, 95% CI: −25.57–55.04; *p* = 0.462), and 36 weeks (Δ = 37.36 m, 95% CI: −4.43–79.16; *p* = 0.078). Consistently, within-group analyses revealed no significant changes in walking distance over time in either hypertensive or normotensive older women (all adjusted *p* > 0.05).

With respect to mobility, improvement in the TUG test showed no time × group interaction (F_2.60,98.84_ = 1.18; *p* = 0.319; η^2^p = 0.03; *small effect*) and no main effect of time (F_2.60,98.84_ = 0.82; *p* = 0.473; η^2^p = 0.02; *small effect*), whereas a significant main effect of group was observed (F_1,38_ = 5.21; *p* = 0.028; η^2^p = 0.12; *moderate effect*). Post hoc analyses demonstrated a significant between-group difference at 12 weeks, with normotensive older women exhibiting faster TUG improvement than hypertensive older women did (Δ = −0.87 s, 95% CI: −1.43–−0.30; *p* = 0.0035), corresponding to a large effect size (*d* = −0.93). No additional between-group differences were detected at baseline, 24 weeks, or 36 weeks, and no significant within-group changes occurred across time.

Regarding lower-body strength–endurance, the 30 s chair stand test revealed a clear time effect (F_2.88,109.2_ = 17.38; *p* < 0.0001; η^2^p = 0.31; *large effect*) in the absence of a time × group interaction (F_2.88,109.2_ = 0.22; *p* = 0.876; η^2^p = 0.01; *small effect*) or a main effect of group (F_1,38_ = 2.44; *p* = 0.127; η^2^p = 0.06; *moderate effect*). At baseline, normotensive older women performed more repetitions than hypertensive older women did (Δ = 2.83 repetitions, 95% CI: 0.23–5.43; *p* = 0.034), reflecting a moderate-to-large between-group effect size (*d ≈* 0.72). Within-group analyses revealed significant improvements relative to baseline in hypertensive older women at 12 weeks (Δ = 4.13, 95% CI: 1.85–6.42; *p* = 0.0003), 24 weeks (Δ = 2.57, 95% CI: 0.55–4.58; *p* = 0.0092), and 36 weeks (Δ = 5.44, 95% CI: 2.74–8.13; *p* < 0.0001). Normotensive older women also exhibited significant increases at 12 weeks (Δ = 3.24, 95% CI: 0.21–6.27; *p* = 0.034) and 36 weeks (Δ = 4.41, 95% CI: 0.74–8.08; *p* = 0.016), with no significant differences between follow-up time points or between groups beyond baseline.

For upper-body muscular strength–endurance, improvement in the arm curl test improved over time (F_2.68,101.9_ = 13.72; *p* < 0.0001; η^2^p = 0.26; *large effect*), without evidence of a time × group interaction (F_2.68,101.9_ = 1.18; *p* = 0.188; η^2^p = 0.04; *small effect*) or a main effect of group (F_1.38_ = 0.20; *p* = 0.611; η^2^p = 0.01; *small effect*). Post hoc analyses indicated significant increases relative to baseline at 12 and 36 weeks in hypertensive older women (Δ = 3.35–5.04 repetitions; adjusted *p ≤* 0.015), as well as at 12 weeks in normotensive older women (Δ = 2.59 repetitions; *p* = 0.036), with no significant between-group differences at any time point.

With respect to upper-limb muscle strength, dominant-hand MIHS had a significant effect on time (F_2.66,101.1_ = 7.51; *p* = 0.0003; η^2^p = 0.17; *large effect*) but not time × group interaction (F_2.66,101.1_ = 0.68; *p* = 0.549; η^2^p = 0.02; *small effect*) or a group effect (F_1,38_ = 0.69; *p* = 0.411; η^2^p = 0.02; *small effect*). Post hoc analyses confirmed that there were no between-group differences at any time point (all *p* ≥ 0.280). Within-group analyses revealed modest but significant increases at 24 weeks compared with baseline in both hypertensive (Δ = −1.50 kg, 95% CI: −2.52 to −0.48; *p* = 0.0026) and normotensive older women (Δ = −1.85 kg, 95% CI: −3.70 to −0.004; *p* = 0.049), with no other significant comparisons.

**Figure 6:**
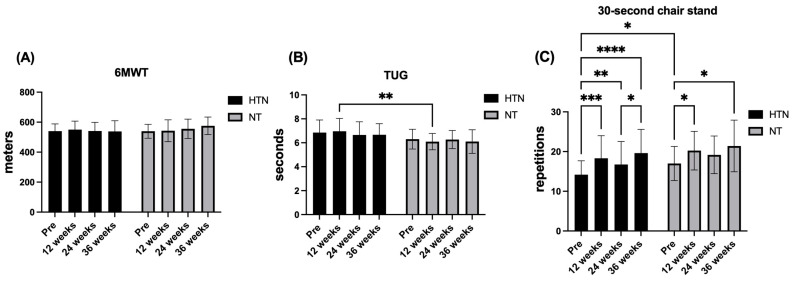
Changes in the (**A**) 6MWT, (**B**) TUG test and (**C**) 30 s chair stand across the intervention period in hypertensive (HTN) and normotensive (NT) older women. The data are presented as the means and (±) standard deviations. * *p* < 0.05, ** *p* < 0.01, *** *p* < 0.001. **** *p* < 0.0001.

**Figure 7:**
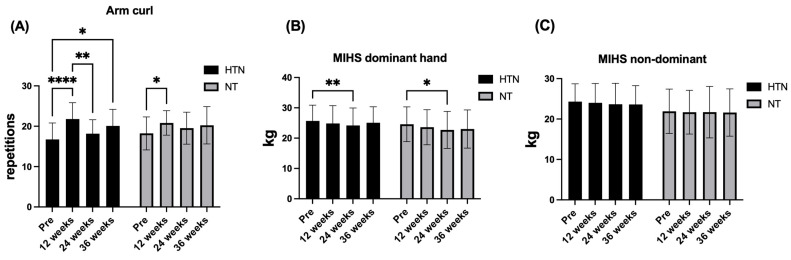
Changes in (**A**) arm curl, (**B**) MIHS dominant hand and (**C**) MIHS nondominant hand across the intervention period in hypertensive (HTN) and normotensive (NT) older women. The data are presented as the means and (±) standard deviations. * *p* < 0.05, ** *p* < 0.01, **** *p* < 0.0001.

Finally, nondominant MIHS remained unchanged across the intervention period. No significant time × group interaction (F_2.51,95.53_ = 0.15; *p* = 0.905; η^2^p = 0.00; *small effect*), main effect of time (F_2.51,95.53_ = 0.62; *p* = 0.578; η^2^p = 0.02; *small effect*), or group (F_1,38_ = 1.85; *p* = 0.182; η^2^p = 0.05; *small effect*) was detected. Post hoc analyses confirmed the absence of between-group differences at baseline and at 12, 24, or 36 weeks (all *p* ≥ 0.150), and within-group analyses revealed no significant changes over time in either group (all adjusted *p* > 0.05). Figure 6 and Figure 7 show the changes in the physical improvement variables in hypertensive and normotensive older women.

## 4. Discussion

The outcomes included significant improvements in blood pressure outcomes (reductions in SBP and DBP), selected cardiometabolic biomarkers (fasting glucose, LDL-c, total cholesterol, and triglycerides, primarily in hypertensive older women), and physical improvement indicators related to muscle strength (arm curl and 30 s chair stand) following MCT combined with a multiprofessional intervention in hypertensive and normotensive older women.

### 4.1. Body Composition

No significant improvements were observed in BFP in either hypertensive or normotensive older women following MCT combined with a multiprofessional intervention. This lack of change suggests that although this intervention effectively improves cardiometabolic and functional parameters, its capacity to induce reductions in adiposity may be limited when training frequency is restricted to three sessions per week and exercise intensity remains predominantly moderate. Under these conditions, the total weekly training load is likely insufficient to generate the sustained caloric deficit required to elicit meaningful reductions in fat mass in older adults [36,37]. In addition, the nutritional component of the intervention, which is valuable for promoting awareness of healthy eating habits on the basis of the Brazilian Dietary Guidelines [31], does not include individualized dietary prescriptions or energy intake control, which are often necessary to achieve measurable changes in adiposity.

In contrast, FFM increased significantly over time in both hypertensive and normotensive older women (12, 24 and 36 weeks), indicating that the intervention was sufficient to stimulate positive adaptations in lean tissue despite the moderate training frequency. These findings suggest that even in the presence of age-related anabolic resistance, which typically attenuates the hypertrophic response to exercise, combined training programs may still promote modest but meaningful gains in FFM when sustained over the medium term. Nevertheless, the absence of between-group differences and the lack of further increases across follow-up time points indicate that higher training intensities, increased volume, or more targeted nutritional strategies, particularly adequate protein intake, may be required to maximize muscle mass accretion in this population [36,37].

The present findings partially align with those of previous studies. Sousa et al. [38] reported significant reductions in BFP in hypertensive older adults after 32 weeks of concurrent training performed three times per week compared with aerobic training alone, supporting the notion that higher training volumes or longer intervention durations are needed to induce fat loss. Conversely, Sousa Junior et al. [39] reported no significant increases in FFM after 12 weeks of walking exercise in adults over 40 years of age with hypertension, and Thompson et al. [40] reported no significant gains in FFM following 8 weeks of concurrent training in older adults with hypertension. Together, these studies suggest that short-term interventions may be insufficient to overcome physiological barriers to muscle mass gain, whereas longer-duration programs, such as the present intervention, may allow lean mass adaptations to emerge even in older populations.

### 4.2. Blood Pressure

Regarding blood pressure outcomes, no significant group × time interaction was observed for either SBP or DBP, indicating that the temporal pattern of change did not differ statistically between hypertensive and normotensive older women. Nevertheless, a robust main effect of group was consistently observed for SBP, with normotensive older women exhibiting lower SBP values than hypertensive older women at all assessed time points. These between-group differences were present at baseline (*d* = 1.33), 12 weeks (*d* = 1.28), 24 weeks (*d* = 1.12), and 36 weeks (*d* = 1.16), reflecting large and stable effect sizes throughout the intervention period and underscoring a persistent separation in SBP between groups over time. In addition to these between-group differences, within-group analyses revealed significant time-related reductions in SBP in both cohorts. Hypertensive older women presented significant decreases in SBP at 12 and 36 weeks relative to baseline, whereas normotensive older women presented significant reductions at the same time points, although with a smaller magnitude. Together, these findings indicate that MCT combined with multiprofessional intervention performed three times per week can elicit clinically meaningful SBP reductions over time while maintaining a clear distinction between groups according to baseline blood pressure status.

For DBP, the response pattern differed from that observed for SBP. No significant main effect of group was detected across the intervention period; however, a significant between-group difference emerged at 12 weeks only, favouring normotensive older women and accompanied by a large effect size (*d* = 0.97). Beyond this time point, DBP values converged between groups, despite the presence of significant within-group reductions—at 36 weeks in hypertensive older women and at 12 weeks in normotensive older women. This transient between-group effect suggests that DBP responses to MCT may be more time sensitive and less stable than SBP, potentially reflecting differences in autonomic regulation and peripheral vascular resistance [41]. The earlier and more pronounced SBP reductions observed in normotensive older women may be partly explained by greater baseline vascular compliance, facilitating faster hemodynamic adaptations to repeated exercise stimuli [41]. In contrast, hypertensive individuals typically present with increased arterial stiffness, endothelial dysfunction, and heightened sympathetic activity, conditions that may require longer exposure to training before substantial blood pressure reductions are achieved [41]. The physiological mechanisms underlying these improvements likely involve increased endothelial nitric oxide bioavailability, increased production of vasoactive substances such as prostaglandins in response to augmented blood flow and high muscle mass activation, and reductions in sympathetic nervous system activity, collectively leading to decreased peripheral vascular resistance [41,42,43]. However, it is important to acknowledge that the potential for unquantified or self-reported changes in antihypertensive medication over the 36-week intervention period could also have contributed to the observed reductions in blood pressure, complicating the direct attribution of these effects solely to the intervention.

These findings are consistent with previous studies reporting significant DBP reductions following MCT in hypertensive older adults compared with inactive controls [19], as well as evidence showing superior DBP reductions with concurrent training relative to aerobic or resistance training alone [44]. In Brazil, Coelho Junior et al. [12] reported significant reductions in DBP in both hypertensive and normotensive older adults following MCT, whereas SBP reductions were observed predominantly in hypertensive participants. The present study extends this evidence by demonstrating large and persistent between-group effects for SBP across all time points (*d* = 1.12–1.33), alongside a transient but large between-group effect for DBP at 12 weeks (*d* = 0.97), highlighting distinct regulatory responses of systolic and diastolic components to exercise training in older women.

From a clinical perspective, the magnitude of SBP reduction observed in hypertensive older women (≈23 mmHg) is substantial and may have important cardiovascular implications. Large-scale meta-analyses indicate that each 10 mmHg reduction in SBP is associated with approximately 20% and 27% lower risks of coronary heart disease and stroke, respectively [45,46]. Accordingly, the observed SBP reductions—together with the large and consistent between-group effect sizes—may translate into a meaningful reduction in cardiovascular risk. Although smaller and less persistent, the DBP changes observed, particularly the large between-group effect at 12 weeks, remain within a range considered beneficial for vascular aging and long-term cardiovascular protection, further supporting the clinical and translational relevance of MCT combined with multiprofessional support in older women.

### 4.3. Biomarkers

In the present study, no significant group × time interaction was identified for LDL-c, indicating that the overall temporal pattern of change did not differ statistically between hypertensive and normotensive older women. However, post hoc analyses revealed significant within-group reductions in LDL-c at 24 and 36 weeks compared with baseline in hypertensive older women, whereas no significant changes were observed in the normotensive group. These time-related reductions were accompanied by moderate between-time effect sizes (*d* = 0.54), suggesting a meaningful magnitude of change despite the absence of an interaction effect. Although these findings should be interpreted cautiously, they are consistent with the meta-analysis by Hejazi et al. [11], which reported reductions in LDL-c following concurrent and resistance-based training protocols lasting 12 weeks or longer. Taken together, the present results suggest that the combination of aerobic and resistance exercise inherent to MCT, when performed three times per week and maintained over a sufficient duration, may be associated with favourable changes in LDL-c, particularly in older women with elevated baseline cardiometabolic risk. Potential mechanisms underlying these adaptations may include increased activity of lipoprotein lipase, facilitating triglyceride-rich lipoprotein hydrolysis, and enhancing hepatic LDL receptor sensitivity, promoting greater clearance of circulating LDL particles [47,48]. Nevertheless, given the exploratory nature of the mechanistic inference in the present study, these pathways should be interpreted as plausible rather than definitive. Nevertheless, the observed reductions in LDL-c, total cholesterol, and fasting glucose, particularly among hypertensive older women, must be interpreted with caution, as unreported or uncontrolled adjustments in lipid-lowering or glucose-regulating medications could have influenced these outcomes.

With respect to HDL-c, no significant time-related changes were observed and no group × time interactions were detected, indicating that HDL-c levels remained largely stable throughout the intervention period. However, a significant main effect of group was consistently present, with normotensive older women exhibiting higher HDL-c concentrations than hypertensive older women at all assessed time points. Post hoc analyses confirmed these persistent between-group differences at baseline and at 12, 24, and 36 weeks (all *p* ≤ 0.017), accompanied by large effect sizes at baseline (*d* = 1.07), 12 weeks (*d* = 0.93), 24 weeks (*d* = 0.78), and 36 weeks (*d* = 0.87). The absence of time-related increases in HDL-c contrasts with findings from Hejazi et al. [11], who reported significant HDL-c elevations following aerobic and concurrent training. This discrepancy may be partly explained by differences in training volume and energy expenditure, as substantial increases in HDL-c are typically associated with exercise programs characterized by increased weekly caloric expenditure and sustained lipid substrate mobilization [49,50]. In the present study, the training frequency (three sessions per week), while sufficient to elicit improvements in other cardiovascular parameters, may not have provided a strong enough stimulus to enhance reverse cholesterol transport and HDL remodelling [51]. In addition, the absence of individualized nutritional strategies, particularly dietary modifications aimed at increasing unsaturated fat and omega-3 fatty acid intake, may further limit HDL responsiveness [52]. Age-related metabolic inflexibility and reduced hepatic apolipoprotein A-I synthesis in older adults may also attenuate the HDL-raising effects of regular physical activity in this population [53].

With respect to total cholesterol, no significant group × time interaction was identified, indicating that the temporal pattern of change did not differ statistically between hypertensive and normotensive older women. Nevertheless, post hoc analyses revealed significant within-group reductions in total cholesterol at 12, 24, and 36 weeks compared with baseline in hypertensive older women, whereas no significant changes were observed in the normotensive group. This pattern suggests a time-related improvement in total cholesterol that was confined to participants with elevated baseline cardiometabolic risk. These findings are consistent with the results reported by Hejazi et al. [11], who reported reductions in total cholesterol following concurrent and strength training interventions, particularly when programs were maintained for 12 weeks or longer. In the present study, the sustained combination of aerobic and resistance exercise over 36 weeks may have contributed to favourable lipid adaptations, potentially through enhanced hepatic LDL receptor activity and improved lipoprotein clearance [48], supported by repeated stimulation of lipoprotein lipase activity and improvements in insulin sensitivity [47]. However, these mechanistic interpretations should be considered plausible rather than definitive. Conversely, the lack of significant changes among normotensive older women may reflect lower baseline cholesterol concentrations, limiting the magnitude of potential improvement as well as interindividual variability and the relatively moderate weekly training volume. Together, these factors may have reduced the likelihood of detecting measurable changes in total cholesterol among individuals closer to normative lipid ranges [54].

Our study did not identify a significant group × time interaction for triglycerides, indicating that the overall temporal pattern did not differ statistically between hypertensive and normotensive older women. However, post hoc analyses revealed a significant increase in triglyceride concentrations at 24 weeks compared with baseline in hypertensive older women, whereas no consistent within-group changes were observed across time in the normotensive group. In addition, a significant difference between 12 and 36 weeks was detected in normotensive older women, suggesting distinct temporal fluctuations between groups. This heterogeneous pattern contrasts with findings from Montrezol et al. [55], who reported significant reductions in triglycerides following a 12-week strength training intervention in hypertensive older adults, but it is consistent with reports by Ruangthai and Phoemsapthawee [56], who reported no significant triglyceride changes after aerobic or resistance training in hypertensive older individuals. Together, these discrepancies highlight the variability in triglyceride responsiveness to exercise interventions, which appears to be strongly influenced by baseline metabolic status, intervention characteristics, and interindividual variability. The transient increase in triglyceride levels observed in hypertensive older women at mid-intervention should be interpreted cautiously. Rather than reflecting an adverse adaptation, this response may be related to short-term alterations in lipid turnover, hepatic VLDL production, or dietary variability, factors that were not directly controlled or measured in the present study. Although classical mechanisms such as increased skeletal muscle lipoprotein lipase activity are typically associated with triglyceride clearance, exercise-induced changes in insulin sensitivity, hepatic lipid metabolism, and fatty acid flux may follow nonlinear trajectories over prolonged interventions [57,58,59]. Consequently, the present findings suggest that triglyceride responses to multicomponent training may be time-dependent and nonmonotonic, particularly in older women with hypertension.

Finally, although favourable time-related changes were observed in selected cardiometabolic biomarkers, particularly LDL-c, total cholesterol, glucose, and blood pressure components, these findings should be interpreted within the context of the study design. The absence of significant group × time interactions for most biomarkers indicates that the temporal patterns of change were broadly similar between hypertensive and normotensive older women, limiting causal inferences regarding differential responsiveness to the intervention. Moreover, the potential influence of uncontrolled factors such as medication use, dietary habits, and habitual physical activity may have contributed to the variability observed in lipid and glucose responses. Accordingly, while several biomarkers exhibited statistically significant and clinically meaningful within-group changes, particularly among hypertensive older women, these results should be viewed as context-dependent adaptations rather than definitive intervention effects. Nevertheless, the overall direction and consistency of the observed changes, especially for LDL-c, total cholesterol, glucose, and systolic blood pressure, are in line with existing evidence supporting the role of regular multicomponent training in promoting metabolic and cardiovascular health in older adults [11,55]. Taken together, these findings contribute to the growing body of literature suggesting that MCT combined with multiprofessional support may represent a viable non-pharmacological strategy for cardiometabolic risk management in older populations, highlighting the need for future studies with tighter control of behavioral and clinical confounders.

### 4.4. Physical Fitness

With respect to the 30 s chair stand test, no significant group × time interaction was observed, indicating that the temporal pattern of change did not differ statistically between hypertensive and normotensive older women. Nevertheless, post hoc analyses revealed significant within-group improvements over time in both groups, with moderate to large effect sizes (ES = 0.69–1.12), suggesting meaningful gains in lower-limb functional strength across the intervention period. These findings differ from those reported by Sousa et al. [38], who reported no significant changes following concurrent or aerobic training in hypertensive older adults across multiple time points (8–32 weeks). In contrast, these findings are consistent with the results of Ruangthai et al. [60], who reported significant improvements in chair stand improvements after concurrent training programs conducted on land and in water among hypertensive older adults in Thailand. Taken together, these discrepancies across studies may reflect differences in intervention duration, exercise content, and participant characteristics. In the present study, the MCT programme combined with multiprofessional intervention, performed three times per week, appeared to have provided a sufficient neuromuscular stimulus to promote improvements in functional lower-body strength. The repeated engagement of large muscle groups, such as the gluteals, quadriceps, and hamstrings, alongside dynamic balance and mobility exercises, may have enhanced motor unit recruitment, firing frequency, and inter- and intramuscular coordination [61]. These neuromuscular adaptations are particularly relevant for preserving and improving lower-limb functional capacity in older adults, with direct implications for independence and fall prevention [62].

Similarly, for the arm curl test, no significant group × time interaction was detected, indicating that the temporal pattern of change was comparable between hypertensive and normotensive older women. Nevertheless, post hoc analyses revealed time-related within-group improvements in both groups, with small to large effect sizes (ES = 0.42–1.23). Significant increases were observed at 12 and 36 weeks in hypertensive older women and at 12 weeks in normotensive older women, suggesting a consistent improvement in upper-body muscular endurance over the intervention period. These findings are consistent with those reported by Son et al. [63], who reported significant improvements in arm curl improvements following 12 weeks of concurrent training in hypertensive older women compared with inactive controls. Similarly, Ruangthai et al. [60] reported significant gains in arm curl improvement among hypertensive older adults, particularly following land-based concurrent training programs. The arm curl test reflects the capacity to sustain repeated elbow flexor contractions while maintaining rhythm and coordination [25]. In this context, the observed improvements may be attributed to the regular inclusion of upper-limb resistance exercises throughout the MCT programme, which likely enhanced neuromuscular efficiency via increased motor unit recruitment, improved firing frequency, and greater inter- and intramuscular coordination [61]. Importantly, although no differential group response was detected, these adaptations may have practical relevance by supporting the upper-limb functional capacity required for daily activities such as carrying groceries or lifting objects, thereby contributing to the maintenance of functional independence in older adults [64].

In contrast, no significant group × time interaction was observed for MIHS in either the dominant or non-dominant hand. However, within-group analyses revealed a modest but statistically significant increase in dominant-hand MIHS at 24 weeks compared with baseline in both hypertensive and normotensive older women, whereas no significant changes were detected at other time points. Importantly, no between-group differences were observed at any assessment, and MIHS in the non-dominant hand remained unchanged throughout the intervention period in both groups. These findings contrast with those reported by Abrahin et al. [65], who reported significant improvements in handgrip strength in both hands among hypertensive older adults following strength training compared with aerobic training. A plausible explanation for the limited responsiveness observed in the present study is that the MCT programme did not include high-intensity or grip-specific exercises capable of generating sufficient mechanical overload on the forearm flexor musculature to induce substantial strength gains [66]. In addition, the predominance of multijoint exercises performed with moderate loads may have reduced the stimulus directed specifically toward the muscles responsible for maximal grip force production [67]. Consequently, although the MCT program was sufficient to elicit transient improvements in dominant-hand MIHS, a training program with greater specificity in handgrip exercises may be needed to achieve sustained or bilateral gains in maximal grip strength.

Similarly, no significant improvements in cardiorespiratory fitness with the 6MWT were observed in either group through MCT combined with multiprofessional intervention, similar to the results reported by Kaholokula et al. [68], in hypertensive older people in Hawaii through a group-based dance intervention, who showed no significant improvements (*p* > 0.05) in the 6MWT compared with the inactive control group. In contrast, compared with the inactive control group, Son, Sung, Cho and Park [63] resulted in significant improvements (*p* < 0.05) in the 6MWT in hypertensive older women through concurrent training. The lack of improvement observed in our study may be related to the reduced aerobic stimulus within the MCT programme. This protocol includes endurance activities. They share session time with flexibility, strength, mobility, motor coordination, and balance exercises [69]. Consequently, the volume and intensity specifically allocated to cardiorespiratory development are lower than those in traditional concurrent training programs, where the session is divided exclusively between strength exercise and aerobic exercise [70]. In our case, the MCT intervention consisted of three moderate-intensity sessions per week, which were likely insufficient to induce the central (increased stroke volume and maximal cardiac output) and peripheral (greater capillary density and mitochondrial content) adaptations necessary to increase submaximal walking capacity [71]. In contrast, Son, Sung, Cho, and Park [63] prescribed exercise intensity on the basis of heart rate reserve, with participants training three times per week for 12 weeks and progressing from 40% to 70%. They reported significant improvements (*p* < 0.05) in the 6MWT in hypertensive older women. These findings underscore the importance of considering not only the inclusion of an aerobic component in MCT programs but also its volume and intensity to elicit detectable improvements in cardiorespiratory capacity [72].

In addition, in the present study, no significant time-related improvements in TUG performance were observed in either hypertensive or normotensive older women, and no group × time interaction was detected. However, a significant main effect of group was identified, with normotensive older women exhibiting faster TUG improvement than hypertensive older women did at 12 weeks (*d* = −0.93), whereas no between-group differences were observed at baseline, 24 weeks, or 36 weeks. This pattern suggests a transient between-group difference rather than a sustained training-induced improvement in functional mobility. These findings are consistent with those reported by Kohn et al. [73], who reported no significant changes in TUG improvement following a tai chi intervention in older adults with hypertension. In contrast, Sarinukul et al. [74] reported significant improvements in TUG performance among hypertensive older women following an aerobic training program compared with inactive controls, highlighting the potential influence of training modality and specificity. The absence of consistent improvements in the TUG test in the present study may be partly explained by the relatively moderate overall training volume, as the MCT programme combined with multiprofessional intervention was delivered three times per week. Moreover, within each session, the time specifically devoted to dynamic balance, agility, and rapid change-of-direction tasks, key components underpinning TUG improvement, was limited. Given that the TUG test requires the integration of speed, postural transitions, and dynamic balance, it is likely that greater task-specific emphasis and higher weekly exposure are necessary to elicit measurable and sustained improvements in this outcome [75]. Therefore, MCT programs aimed at improving functional mobility in older adults may benefit from increasing both the volume and specificity of balance- and mobility-focused exercises.

### 4.5. Strengths and Limitations

The limitations of this study were as follows: (i) The inclusion of only older women along with longer-term monitoring to examine the maintenance of benefits. (ii) The challenge in rigorously controlling and precisely quantifying external factors, such as medication adherence, dietary variability, and physical activity independent of the intervention. Although participants were instructed to maintain their habitual routines and any relevant changes were recorded, the absence of systematic, detailed reporting on the specific types and dosages of antihypertensive or lipid-lowering medications and whether these were adjusted during the 36-week period constitute a significant potential confounding factor. Given that medication adjustments are common in older hypertensive patients, the observed changes in blood pressure and cardiometabolic biomarkers cannot be unequivocally attributed solely to the intervention, as pharmacological modifications may have contributed to or interacted with these outcomes. These variables may act as confounding factors, influencing the observed results in biomarkers and blood pressure, and represent an inherent limitation of the quasi-experimental design in a real-world setting. (iii) Potential selection bias, as participants who volunteer may have been more motivated or physically capable than the general older people, which could restrict the applicability of our results. (iv) The absence of follow-up measurements for biomarkers and physical improvement variables after the intervention. (v) The quasi-experimental design, which, by not being a randomized controlled trial, introduces the potential for selection bias and limits the strength of causal inferences. While this design was chosen to evaluate the intervention in a practical community setting, we acknowledge its inherent limitations in controlling all confounding variables and recommend that future randomized controlled trials build upon these findings; additionally, the most critical limitation is the absence of a control group without intervention. Without a comparison to usual care or an attention-control group, it is difficult to attribute the observed improvements—even the statistically significant changes in blood pressure and lipids—specifically to the intervention. While this design was chosen for its feasibility in a community setting and to provide exploratory insights into differential responses between hypertensive and normotensive women, serving as preliminary data for the future, more rigorously controlled trials [76], alternative explanations, such as regression to the mean, seasonal variation, repeated testing effects, and the Hawthorne effect (attention bias), remain plausible contributors to the observed temporal changes and cannot be definitively ruled out without a true control group. This fundamental design constraint should be explicitly recognized as the primary threat to the internal validity of our findings. (vi) Although the sample size was adequately powered for our primary outcomes on the basis of a priori calculations, it is possible that for some secondary outcomes or more subtle interactions, a larger sample might have revealed statistically significant effects. Therefore, the interpretation of non-significant findings should consider the reported ES in conjunction with potential statistical power limitations for these specific variables. (vii) The primary reliance on the RPE for guiding training intensity, while beneficial for individualization and safety in older populations, may limit the exact replicability of the protocol by other researchers and hinder direct comparisons with studies employing more objective intensity measures (e.g., %1RM or %HRmax). Future research could explore the integration of both perceptual and objective intensity monitoring for enhanced precision. Finally, an additional limitation relates to the observational nature of the comparison between groups, which were defined according to baseline blood pressure status rather than by random allocation. Consequently, between-group differences over time should not be interpreted as causal effects attributable to the intervention. Moreover, several alternative explanations for the changes observed during the intervention period cannot be ruled out, including regression to the mean, particularly in the hypertensive group, seasonal variations, potential adjustments in pharmacological treatment, and increased health awareness resulting from participation in the study. These factors may have contributed, at least in part, to the time-related changes detected and should be considered when interpreting the findings.

The strengths of this study include (i) a longitudinal and experimental design spanning 36 weeks with four assessment points (baseline, 12, 24, and 36 weeks), allowing the characterization of time-related changes in multiple health outcomes; (ii) the inclusion of clinically relevant subgroups defined by baseline blood pressure status (hypertensive and normotensive older women), enabling a detailed descriptive comparison of trajectories within distinct cardiovascular risk profiles; (iii) the use of standardized and validated assessments commonly applied in exercise and aging research, supporting the robustness and external validity of the findings; (iv) the comprehensive evaluation of cardiometabolic health through the combined assessment of blood pressure, biochemical markers, body composition, and physical improvement outcomes within a real-world, community-based intervention setting.

### 4.6. Practical Applications

(i) Time-efficient options: MCT combined with a multiprofessional intervention performed three times per week may represent a time-efficient strategy to maintain and improve selected components of muscular improvement, particularly lower- and upper-limb functional strength (e.g., chair stand and arm curl), in both hypertensive and normotensive older women.

(ii) Blood pressure benefits: Clinically meaningful reductions in systolic and diastolic blood pressure were observed over time, particularly in hypertensive older women, supporting the use of MCT as a non-pharmacological strategy to support blood pressure control in older populations. These findings reinforce the relevance of structured exercise programs as part of comprehensive cardiovascular risk management.

(iii) Specificity for mobility and aerobic capacity: The absence of significant improvements in walking capacity and mobility speed (6MWT and TUG) suggests that higher weekly frequency, greater aerobic volume, or more task-specific motor practice may be required within MCT programs to elicit measurable adaptations in these outcomes.

(iv) Comprehensive cardiovascular monitoring: The inclusion of cardiometabolic biomarkers and blood pressure assessments alongside functional measures allows for a more comprehensive evaluation of exercise-related adaptations, particularly in hypertensive older adults. This multidimensional approach may help clinicians and practitioners better track health-related responses to exercise beyond improvement outcomes alone.

## 5. Conclusions

The present findings suggest that MCT combined with multiprofessional interventions may represent a feasible and time-efficient approach to improving cardiovascular health and functional capacity in older Brazilian women. Over the 36-week intervention, time-related improvements in systolic and diastolic blood pressure, selected cardiometabolic biomarkers (fasting glucose, LDL-c, total cholesterol, and triglycerides), and functional improvement measures related to muscular endurance and strength (arm curl and 30 s chair stand) were observed, particularly among hypertensive participants. However, the absence of significant group × time interactions and the stability of several outcomes underscore the need for cautious interpretation. Collectively, these results support the inclusion of MCT within community and clinical health programs as a complementary strategy for maintaining cardiovascular and functional health in older women while highlighting the importance of intervention duration, exercise specificity, and individualized support.

## Figures and Tables

**Table 1:** Baseline characteristics of the groups analysed.

	Hypertensive Older Women (*n* = 23)	Normotensive Older Women (*n* = 17)	*p* Value
Age (years)	69.7 ± 7.21	71.3 ± 5.92	0.12
BMI (kg/m^2^)	29.8 ± 6.84	26 ± 3.66	0.06
SBP (mmHg)	157.2 ± 14.6	126.7 ± 16.3	0.00 *
DBP (mmHg)	86.3 ± 11.4	75.4 ± 11.5	0.10

Note: Data are expressed as the mean and (±) standard deviation; BMI = body mass index; SBP = systolic blood pressure; DBP = diastolic blood pressure; mmHg = millimeters of mercury; kg/m^2^ = kilograms divided in square meters; * = significant difference (*p* < 0.05).

**Table 2:** Periodization scheme of the multicomponent training intervention.

Program	Physical Exercise	Training Session	Week Freq.	Session Time	Load	Set	Rep.	Rest	Cadence
MCT Dosage 12 weeks, 24 weeks, and 36 weeks.	Inside	A (9 exercises)	3	≈60 min	RPE	2	40 seg	20 seg	4-0-2-0
		B (8 exercises)	3	≈60 min	RPE	3	30 seg	30 seg	Slowly 1-0-1-0
	Outside	A (6 exercises)	3	≈60 min	RPE	2	40 seg	20 seg	4-0-2-0
		B (7 exercises + 10 min walk)	3	≈60 min	RPE	3	30 seg	45 seg	1-concentric-1-eccentric

Note: MCT = multicomponent training; Rep. = repetitions; RPE = Rating of Perceived Exertion scale; Inside: A = leg press, leg extension, leg curl, chest press, lat pulldown, seated row, overhead press, abdominal crunch (machine), and back extension; B = hack squat, dumbbell row, dumbbell bench press, dumbbell shoulder press, bicep curl, triceps extension, calf raises, and plank (modified on knees or forearms); and outside: A = chair squats/sit-to-stands, resistance band rows (standing), incline push-ups (against a wall or sturdy surface), standing marching/high knees (controlled), single leg stand (with support), and bird-dog; B = walking lunges (partial movement), resistance band overhead press, gluteal bridges, side leg raises (standing), arm circles (forward and backwards), calf raises (standing), toe taps (on a small step/curb) and 10 min brisk walk.

## Data Availability

The authors confirm that the data supporting the findings of this study are available within the article.
